# Neuronal MHC-I complex is destabilized by amyloid-β and its implications in Alzheimer’s disease

**DOI:** 10.1186/s13578-023-01132-1

**Published:** 2023-09-29

**Authors:** Min-Seok Kim, Kwangmin Cho, Mi-Hyang Cho, Na-Young Kim, Kyunggon Kim, Dong-Hou Kim, Seung-Yong Yoon

**Affiliations:** 1 ADEL Institute of Science & Technology (AIST), ADEL, Inc., Seoul, Korea; 2 Department of Brain Science, Asan Medical Center, University of Ulsan College of Medicine, Seoul, Korea; 3 Department of Convergence Medicine, Convergence Medicine Research Center/Biomedical Research Center, Asan Medical Center, University of Ulsan College of Medicine, Seoul, Korea

**Keywords:** Alzheimer’s disease, MHC-I, β2-microglobulin, NCAM1, High affinity peptide, Amyloid-β

## Abstract

**Backgrounds:**

The expression of major histocompatibility complex I (MHC-I) in neurons has recently been shown to regulate neurite outgrowth and synaptic plasticity. However, its contribution to neurodegenerative diseases such as Alzheimer's disease (AD) remains largely unknown.

**Methods:**

In this study, we investigated the relationship between impaired MHC-I-β2M complex and AD in vitro and human AD samples. Interaction between protein was identified by liquid chromatography-tandem mass spectrometry and confirmed by immunoprecipitation. Single-chain trimer of MHC-I-β2M was generated to study the effect of stabilization of MHC-I-β2M complex on NCAM1 signaling.

**Results:**

MHC-I is destabilized in the brains of AD patients and neuronal cells treated with oligomeric β-amyloid (Aβ). Specifically, Aβ oligomers disassemble the MHC-I-β2-microglobulin (β2M) complex, leading to reduced interactions with neural cell adhesion molecule 1 (NCAM1), a novel interactor of neuronal MHC-I, and decreased signaling. Inhibition of MHC-I-β2M complex destabilization by non-dissociable MHC-I-β2M-peptide complex restored MHC-I-NCAM1 signaling in neuronal cells.

**Conclusions:**

The current study demonstrated that disruption of MHC-1-NCAM1 signaling by Aβ induced disassembly of MHC-I-β2M complex is involved in the pathophysiology of AD. Moreover, our findings suggest modulation of MHC-I stability may be a potential therapeutic target for restoring synaptic function in AD.

**Supplementary Information:**

The online version contains supplementary material available at 10.1186/s13578-023-01132-1.

## Introduction

Major histocompatibility complex I (MHC-I), a functional trimeric complex composed of MHC-I heavy chains, β_2_-microglobulin (β_2_M), and a short antigenic peptide, participates in the adaptive immune system by presenting intracellular antigenic peptides to cytotoxic T lymphocytes [1]. Although MHC-I is expressed in all the nucleated cells and was extensively studied in immune systems, less was known about MHC-I in the nervous system because brain was thought to be an immune-privileged organ. However, it began to be reported that MHC-I is also expressed in neurons of developing and adult brains [2], and is involved in the regulation of neurite outgrowth and synaptic plasticity in the central nervous system (CNS) [3]. MHC-I may also play a role in the regulation of initial synapse connections during CNS development [4–6], and in the regulation of synaptic density in the visual cortex and GABAergic synaptic density in cultured neurons [7]. Despite MHC-I’s active role in CNS, little is known about its pathophysiological roles in various neurological and psychological diseases.

Although several meta-analyses of genetic risk factors for Alzheimer’s disease (AD) have suggested that MHC-I alleles, specifically HLA-A2.1 [8] and HLA-B7 [9], are associated with AD pathology, the relationship between MHC-I with AD remains unclear because of contradictory reports in differential populations [10]. Amyloid-β (Aβ) peptide is a major pathological factor in synaptic dysfunction. Specifically, oligomeric Aβ dysregulates activity of several synaptic receptors. α-amino-3-hydroxy-5-methyl-4-isoxazolepropionic acid (AMPA) and n-methyl-d-aspartate (NMDA) receptors, whose function and trafficking are regulated by synaptic MHC-I [11], are, in turn, negatively regulated by Aβ peptide in AD [12, 13]. Additionally, PirB which is a MHC-I receptor expressed in synapses of hippocampal neurons, and is involved in synaptic plasticity during CNS development [14–17], was identified as an amyloid-β (Aβ) receptor and has been implicated in AD pathology mediated by dysregulated synaptic plasticity with cofilin activation [18]. These findings suggest that MHC-I may have a putative role in AD pathophysiology.

We here addressed this point and revealed that Aβ destabilizes MHC-I complex resulting in decrease of NCAM-1 signaling but restoring MHC-I complex reverses its signaling in Aβ-treated neuronal cells.

## Materials and methods

### Human brain tissue

Medial temporal gyri from eight patients with AD and seven age- and sex-matched controls were provided by the Netherlands Brain Bank (Additional file 3: Table S1). AD pathological staging was based on the Braak staging system [19]. The hippocampus and cortex from the brains of normal controls and AD patients were analyzed by qRT-PCR, western blotting, synaptosome purification, and immunoprecipitation.

### DNA constructs

The DNA construct expressing the SCT of HLA-A2.1 (TAX-HLA-A2.1-hβM) was the kind gift of Dr. Ted H. Hansen (Washington University School of Medicine, St. Louis, MO, USA). cDNAs encoding HLA-A2.1-WT and HLA-A2.1-SCT were amplified by polymerase chain reaction (PCR) and cloned into the mammalian expression vector pcDNA3.1 (Invitrogen). Human NCAM1 cDNA was amplified by PCR from single-stranded cDNA synthesized from total RNA of normal human aged brain with a ReverTra Ace^®^ qPCR RT kit (Toyobo Co., Ltd., Osaka, Japan), according to the manufacturer’s protocol, and cloned into pcDNA3.1 vector. For N-terminus HA-tagging, a HA-Tag sequence was inserted behind the signal sequence region of NCAM1 by PCR.

### Cell culture and isolation of primary mouse cortical neurons

SH-SY5Y cells were maintained in Dulbecco’s modified Eagle’s medium (DMEM; Thermo Fisher Scientific Inc., Waltham, MA, USA) supplemented with 10% fetal bovine serum (FBS; Thermo Fisher Scientific Inc.), and incubated at 37 ℃ in an atmosphere containing 5% CO_2_. Cultures of primary cortical neurons were prepared from the brains of embryonic-day-16 pups, as described [20]. Briefly, the cerebral cortices were dissected in cold calcium- and magnesium-free Hank’s balanced salt solution and incubated in 0.125% trypsin solution for 15 min at 37 ℃. Trypsin was inactivated by the addition of DMEM containing 20% FBS, and cortical tissue was dissociated by repeated trituration using a Pasteur pipette. The cell suspensions were diluted in Neurobasal medium supplemented with B-27 components (Thermo Fisher Scientific Inc.), and seeded onto plates coated with poly-D-lysine (MilliporeSigma, Burlington, MA, USA) and laminin (1 mg/mL; Thermo Fisher Scientific Inc.). Neurons were maintained at 37 ℃ in a humidified 5% CO_2_ environment.

### Immunocytochemistry

Primary cortical neurons (2 × 10^5^ cells) were plated onto 18 mm coverslips (Paul Marienfeld GmbH & Co. KG, Lauda-Königshofen, Germany) coated with 0.05 mg/mL poly-D-lysine and 1 µg/mL laminin (MilliporeSigma). The GFP construct was cotransfected with mock, wild-type (WT), or single chain trimer (SCT) HLA-A2.1 constructs into primary cortical neurons at 3 div using Lipofectamine 2000 (Thermo Fisher Scientific Inc.) according to the manufacturer’s protocol. After 2 h, the cells were treated with Aβ oligomerfor 48 h, fixed with 4% paraformaldehyde, and mounted onto slides for imaging. To assess the co-localization of MHC-I and NCAM1 or MHC-I and β_2_M on primary neurons in the presence or absence of Aβ oligomer treatment, the cells were fixed and permeabilized with PBS containing 0.5% Triton X-100 for 10 min and incubated with blocking buffer (10% NGS, 5% bovine serum albumin [BSA], and 0.5% Tween 20 in PBS) for 1 h. The cells were subsequently incubated with primary anti-mouse MHC-I(B22-249.R1, Thermo Fisher Scientific Inc), anti-NCAM1(Proteintech) and anti-β_2_M(Abcam) antibodies overnight at 4 ℃, washed three times with PBS, and incubated with secondary Alexa fluor 488/594-conjugated anti-mouse or anti-rabbit antibody for 1 h at RT. The cells were again washed three times with PBS, stained with Hoechst 33,342 (Thermo Fisher Scientific Inc.) for 10 min, again washed with PBS, and mounted onto slides.

### Image analysis

Neurons and brain sections were examined with an LSM 780 confocal microscope (Carl Zeiss AG, Jena, Germany), and images were processed with ZEISS ZEN software (Carl Zeiss AG). For the quantitative analysis of neurite outgrowth, low-power magnified (10 ×) images of GFP-positive neurons were acquired from 20 different fields of view per sample and analyzed using MetaMorph software (Molecular Devices, LLC, San Jose, CA, USA). For the quantitative analysis of cells co-expressing MHC-I and β_2_M or MHC-I and NCAM1, images of cells positive for both were acquired from 10 different field of view per sample from six mice.

### Coimmunoprecipitation and western blot analyses

For coimmunoprecipitation, purified synaptosomes (human/mouse), brain tissue (human/mouse), SH-SY5Y cells expressing NCAM1-HA, and cultured mouse cortical neurons were lysed with 1% digitonin (Millipore Sigma) containing 50 mM HEPES, 100 mM NaCl, 10 mM CaCl_2_, and 5 mM MgCl_2_ (pH 7.6) supplemented with a protease inhibitor cocktail (Millipore Sigma) for 30 min at 4 ℃. The lysates were precleared with protein G-sepharose (GE Healthcare, Little Chalfont, UK) for 1 h at 4 ℃. Immunoprecipitation was performed by overnight incubation with anti-human MHC-I antibody (W6/32; Thermo Fisher Scientific Inc.), anti-mouse MHC-I antibody (2G5; Bio-Rad Laboratories, Inc., Hercules, CA, USA), or anti-HA antibody (11867423001, Roche Diagnostics Corporation, Indianapolis, IN, USA) at 4 ℃. Immune complexes were purified using protein G-sepharose followed by three washes with 0.1% digitonin. Immunoprecipitated proteins were eluted by boiling in sodium dodecyl sulfate (SDS) sample buffer. Immunoprecipitated samples and 5% of the lysate preparations were electrophoresed on SDS-PAGE gels, which were stained with Coomassie blue or subjected to immunoblotting.

For western blot analyses, the protein lysates described above were homogenized in 1% Triton X-100 in PBS and incubated for 30 min at 4 ℃. The preparations were centrifuged at 13,000 g for 15 min at 4 ℃, and the protein concentrations in the supernatant were measured by Bradford assays. Protein samples were mixed with 5X SDS sample buffer [60 mM Tris–HCl (pH 6.8), 2% (w/v) SDS, 25% (v/v) glycerol, 14.4 mM (v/v) β-mercaptoethanol, and bromophenol blue], boiled for 5 min, and stored at – 20 ℃. The proteins were separated by 10% SDS–polyacrylamide gel electrophoresis (PAGE), transferred to polyvinylidene difluoride membranes (pore size, 0.2 mm; Bio-Rad Laboratories, Inc.), and blocked with 5% (v/v) skim milk in 0.1% (v/v) Tween-20 in PBS (PBS-T) for 1 h at RT. The blots were incubated with primary antibodies overnight at 4 ℃, washed three times in PBS-T buffer, and incubated with horseradish peroxidase-conjugated secondary antibodies for 1 h. The immunoblots were visualized using ECL reagents (Thermo Fisher Scientific Inc.). The primary antibodies used in the western blotting analyses were anti-human MHC-I (NBP2-66946, Novus Biologicals, LLC, Littleton, CO, USA); anti-β_2_M (Abcam plc, Cambridge, UK); anti-NCAM1 (14255-1-AP, Proteintech Group, Inc., Rosemont, IL, USA); anti-β-actin (MilliporeSigma); anti-protein disulfide isomerase (Thermo Fisher Scientific, Inc.); anti-mouse MHC-I (OX-18; Abcam plc); anti-PSD95, anti-synaptophysin, anti-glyceraldehyde 3-phosphate dehydrogenase (MilliporeSigma); and anti-ERp57, anti-tapasin, anti-TAP, anti-pCREB, anti-CREB, anti-c-fos, and anti-BDNF (Santa Cruz Biotechnology, Inc., Dallas, TX, USA). The band intensities were measured and analyzed with ImageJ software (https://imagej.nih.gov/ij/).

### Synaptosomes

Synaptosomes were purified as described [21]. Briefly, frozen human or mouse brain tissues were homogenized in isotonic sucrose buffer (0.32 M sucrose, 1 mM ethylenediaminetetraacetic acid, 5 mM Tris, pH 7.4) supplemented with protease inhibitor cocktail (MilliporeSigma) with 20 strokes of a Dounce homogenizer. The homogenates were centrifuged at 1,000 × *g* for 10 min at 4 ℃. The supernatants were loaded onto Percoll (GE Healthcare) gradients (3%, 10%, 15%, and 23% in isotonic sucrose buffer) and ultracentrifuged at 31,000 × *g* for 5 min at 4 ℃. All fractions were collected and analyzed by western blotting. For immunoprecipitation, the synaptosome pellets were lysed with 1% Triton X-100 in PBS supplemented with protease inhibitor cocktail for 30 min at 4 ℃. The lysates were incubated with human anti-MHC-I antibodies [W6/32 (Thermo Fisher Scientific Inc.) or HC10 (OriGene Technologies, Inc., Rockville, MD, USA)] overnight at 4 ℃, and the immunoprecipitates were purified on protein G-sepharose. The boiled eluents were analyzed by western blotting. To identify novel proteins that interacted with MHC-I, synaptosomes were prepared from the hippocampal region (1 g) of normal aged human brain.

### Bio-layer interferometry assay

Interactions between NCAM1 and MHC-I–β2M were analyzed using the BLItz^®^ system (Pall ForteBio LLC, Fremont, CA, USA), which measures bio-layer interferometry. To analyze the binding of NCAM1 and MHC-I–β2M, 100 nM of recombinant his-tagged NCAM1 protein (Abcam plc) was immobilized on a Ni–NTA biosensor (Pall ForteBio LLC), and the immobilized protein was incubated with the recombinant biotinylated stable form of MHC-I–β2M (HLA-A2.1 allele, MBL International Corporation, Woburn, MA, USA), diluted in PBS to concentrations of 0, 50, 100, 250, 500, and 1000 nM. Ligands (NCAM1 or MHC-I–β2M) were loaded onto biosensors for 120 s and washed. Binding of the ligand-analyte was monitored for 120 s, and the binding affinity was calculated by the BLItz^®^ system.

### Flow cytometry

Cell-surface MHC-I expression was analyzed by flow cytometry. SH-SY5Y cells were incubated with oAβ for 24, 48, and 72 h. The cells were harvested, washed twice in cold PBS containing 1% BSA, and incubated with W6/32 or anti-HA antibody for 1 h at 4 ℃. Labeled cells were washed twice in cold PBS containing 1% BSA and stained with FITC-conjugated goat anti-mouse IgG for 1 h at 4 ℃. As a negative control, the cells were incubated with normal mouse IgG. In each sample, a total of 10,000 gated events were collected by FACSCanto II flow cytometer (BD Biosciences, San Jose, CA, USA) and analyzed with FACSDiva software (BD Biosciences).

### Aβ peptide

Aβ oligomers were prepared as described [20]. Briefly, lyophilized Aβ_1-42_ peptides (4014447, Bachem AG, Bubendorf, Switzerland) were dissolved in dimethyl sulfoxide (DMSO) to a final concentration of 500 µM and diluted in DMEM to a final concentration of 1 µM. Oligomeric Aβ was prepared by incubating diluted monomeric Aβ for 24 h at 4 ℃. Untreated control are treated 0.2% DMSO.

### Quantitative real-time PCR

Total RNA was isolated from brain tissues of normal individuals and AD patients, from wild-type mouse brain tissues, and from Aβ-treated SH-SY5Y cells using TRIzol (Thermo Fisher Scientific Inc.). Single-stranded cDNA was synthesized with a ReverTra Ace^®^ qPCR RT kit according to the manufacturer’s protocol. Quantitative reverse transcription-PCR (qRT-PCR) was performed using a LightCycler^®^ 480 II (Roche Diagnostics Corporation) with iQ^™^ SYBR^®^ Green Supermix (Bio-Rad Laboratories, Inc.). The primers used for qRT-PCR included those for HLA-ABC (forward, 5′-GCCTACGACGGCAAGGATTAC-3′; reverse, 5′-GGTGGCCTCATGGTCAGAGA-3′), and glyceraldehyde 3-phosphate dehydrogenase (forward, 5ʹ-AATCCCATCA CCATCTTCC-3′; reverse, 5′-GGACTCCACG ACGTACTCA-3′).

### Glycosylation analysis

Aged normal and AD brain tissues were homogenized in tissue extract buffer (1% Triton X-100, 50 mM Tris–HCl [pH 8.0], 150 mM NaCl, and 5 mM ethylenediaminetetraacetic acid supplemented with protease inhibitor cocktail). The lysates were incubated with W6/32 antibody for 2 h at 4 ℃ and then with protein G-sepharose. To analyze MHC-I glycosylation, immunoprecipitates were digested at 37 ℃ for 1 h with 3 mM endoglycosidase-H (Roche Diagnostics Corporation) or peptide-n-glycosidase F (New England BioLabs, Inc., Ipswich, MA, USA) according to the manufacturers’ protocols. The beads were boiled in SDS sample buffer for 10 min and analyzed by immunoblotting.

### Cell-surface protein biotinylation

To investigate the changes in cell-surface MHC-I structure induced by Aβ, SH-SY5Y cells were washed twice in cold PBS and incubated in cold PBS containing 10 mM sulfo-NHS-SS-biotin (Thermo Fisher Scientific Inc.) for 30 min at 4 ℃. The cells were washed twice in cold PBS and quenched with 50 mM Tris–Cl (pH 8.0) before treatment with Aβ for 1 h at 4 ℃. The Aβ-treated cells were lysed in 1% Triton X-100 in PBS supplemented with protease inhibitor cocktail (MilliporeSigma). The lysates were immunoprecipitated with anti-MHC-I antibodies (W6/32, which recognizes the MHC-I-β_2_M complex; and HC10, which recognizes free MHC-I) or streptavidin-agarose (Thermo Fisher Scientific Inc.). Culture media were also collected and immunoprecipitated with the anti-β_2_M antibody. The immunoprecipitates were analyzed by western blotting.

### In-gel digestion

The SDS-PAGE gels were sliced and chopped into small particles with a clean blade and destained with 200 mM ammonium bicarbonate (ABC) and 50% acetonitrile. The gel particles were dehydrated with 100% acetonitrile and washed with 50 mM ABC. The proteins in the gel were reduced with dithiothreitol at 70 ℃ for 20 min and alkylated with iodoacetamide at RT for 1 h. After washing with 50 mM ABC and 100% acetonitrile, the proteins in the gel were digested with sequencing grade trypsin (Promega Corporation, Madison, WI, USA) on resin at 37 ℃ overnight in digestion buffer (100 mM Tris pH 8.0 and 2 mM CaCl_2_), and the tryptic peptides were desalted using Sep-PAK C18 cartridges (Waters Corporation, Milford, MA, USA) and dried under vacuum in a SpeedVac.

### LC–MS/MS and sequence database analyses

Peptides were separated using the Dionex^™^ UltiMate^™^ 3000 RSLCnano system (Thermo Fisher Scientific Inc.). Tryptic peptides from the bead columns were reconstituted with 0.1% formic acid and separated on a 50 cm Easy-Spray^™^ column of inner diameter 75 μm packed with 2 μm C18 resin (Thermo Fisher Scientific Inc.) for over 120 min (300 nL/min) using a 0–45% acetonitrile gradient in 0.1% formic acid at 50 ℃. The LC system was coupled to a Q Exactive mass spectrometer with a nano-ESI source. The mass spectra were acquired in a data-dependent mode with an automatic switch between a full scan with five data-dependent MS/MS scans. The target value for the full-scan MS spectra was 3,000,000, with a maximum injection time of 120 ms and a resolution of 70,000 at m/z 400. The ion target value for MS/MS was set to 1,000,000 with a maximum injection time of 120 ms and a resolution of 17,500 at m/z 400. Repeated peptides were dynamically excluded for 20 s. The resulting raw files were processed using MaxQuant (version 1.5.2.8), and the sequences were compared with those in the *Homo sapiens* database [organism ID: 9606, 71,567 entries, UniProt (http://www.uniprot.org/)]. The search parameters were set at default, including cysteine carbamidomethylation as a fixed modification; n-terminal acetylation, methionine oxidation, and phospho-serine, -threonine, and -tyrosine as variable modifications; and di-glycine modification at a lysine residue with two miscleavages. Peptide identification was based on a search with an initial mass deviation of the precursor ion of up to 10 ppm, and the allowed fragment mass deviation was set at 20 ppm. The resulting out files from MaxQuant were processed through Scaffold software (version 4.4.1.1; Proteome Software, Inc., Portland, OR, USA) for further statistical analyses and visualization.

### Statistical analysis

Quantitative data were analyzed statistically using GraphPad Prism 7 software (GraphPad Software, Inc., La Jolla, CA, USA). All data are presented as the mean ± the standard error of the mean (SEM), and analyzed by unpaired two-tailed Student’s t-tests or by one-way or two-way ANOVA with Tukey’s post-hoc comparisons tests. *P-*values < 0.05 were considered statistically significant. All experiments were repeated at least three times, and no data were excluded.

## Results

### Aβ-mediated downregulation of MHC-I stability in AD pathology

**Fig. 1:**
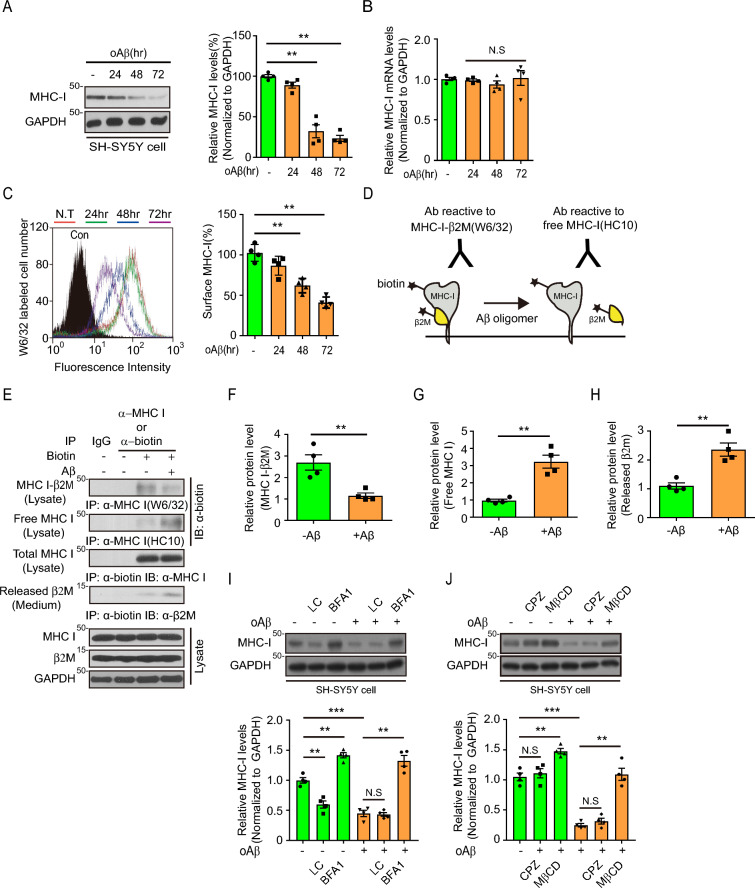
Oligomeric amyloid-β (Aβ) downregulated expression and structure of MHC-I–β_2_M complex. **A** Immunoblot analyses of MHC-I expression levels in Aβ oligomer (oAβ)-treated SH-SY5Y cells (left) The total MHC-I levels were normalized to those of GAPDH (**B**; n = 4, one-way ANOVA with Tukey’s post-hoc comparisons test) (right). **B** MHC-I mRNA levels in oAβ-treated SH-SY5Y cells (n = 4, one-way ANOVA with Tukey’s post-hoc comparisons test). **C** Surface MHC-I was labeled with the W6/32 antibody, analyzed by flow cytometry (n = 4, one-way ANOVA with Tukey’s post-hoc comparisons test). Representative histogram (left) and quantification of fluorescence intensity (right) of surface MHC-I–β_2_M complex. **D–H** Schematic representations of the structural changes in MHC-I–β_2_M complex detectable by different antibodies (W6/32 and HC10) and the release of surface β_2_M by oAβ-treated SH-SY5Y cells (**D**). Immunoprecipitation analysis of Aβ-induced structural changes in surface MHC-I–β_2_M complex (**E**; n = 4). The levels of surface MHC-I–β_2_M complex (**F**) and surface-free MHC-I (**G**) were normalized to total surface MHC-I levels. The levels of released β_2_M were normalized to those of total β_2_M (**H**). The data were analyzed by unpaired two-tailed Student’s t-test (**F**–**H**). **(I, J)** Immunoblot analyses of MHC-I levels in oAβ-treated SH-SY5Y cells in the presence or absence of the proteolytic inhibitors lactacystin (LC) and bafilomycin A1 (BFA1); n = 4 (**I**), or the endocytosis inhibitors chlorpromazine (CPZ) and methyl-β-cyclodextrin (MβCD); n = 4 (**J**). Total MHC-I levels were normalized to the levels of actin. The data were analyzed by one-way ANOVA with Tukey’s post-hoc comparisons test (**I**, **J**). The data are presented as the mean ± SEM (N.S, not significant; *P < 0.05, **P < 0.001, ***P < 0.005)

We first assessed whether Aβ affects MHC-I. SH-SY5Y cells were treated with Aβ oligomers for various time periods, and MHC-I levels were assessed by immunoblotting. Interestingly, Aβ treatment markedly reduced MHC-I levels (Fig. 1A) but MHC-I mRNA levels were unaffected (Fig. 1B), indicating that Aβ-mediated MHC-I downregulation occurred at the protein level. Since the protein stability, especially cell surface quality control, of MHC-I is mainly determined by its complex with antigen peptide and β2M [22], we checked the cell surface level of MHC-I-β2M complex, total MHC-I, with fluorescence-activated cell sorting (FACS) using W6/32 which detects only total human MHC-I, not free MHC-I unbound to β2M. Cell surface level of total MHC-I complexed with β2M was decreased by oligomeric Aβ treatment in a time-dependent manner (Fig. 1C), suggesting dissociation of β2M from MHC-I complex by Aβ oligomers. To investigate this point further, biotinylated cell-surface MHC-I-β2M complexes on SH-SY5Y cells treated with oligomeric Aβ for a short time were purified with specific monoclonal antibodies, either W6/32 or HC10, which recognizes the β2M-free human MHC-I [23, 24] to detect conformational changes in MHC-I-β2M by Aβ oligomers (Fig. 1D). The levels of biotinylated MHC-I-β2M complex reacting with W6/32 were reduced by 60% (Fig. 1E, F) in line with the FACS result (Fig. 1C), whereas the levels of biotinylated free heavy chain of MHC-I reacting with HC10 were increased threefold in Aβ-treated SH-SY5Y cells (Fig. 1E, G), suggesting that Aβ induced dissociation of cell surface MHC-I-β2M complex. This Aβ-mediated destabilization of cell-surface MHC-I-β2M complex was confirmed by the increased extracellular release of β2M into the culture medium from MHC-I–β2M complex (Fig. 1E, H). Because the reduced stability of surface MHC-I–β2M complex accelerates its endocytosis [25], We investigated the intracellular pathways associated with Aβ-mediated MHC-I downregulation. The change of total MHC-I levels was examined after Aβ treated SH-SY5Y cells were treated with or without proteolysis inhibitors, either the proteasome inhibitor lactacystin or the lysosome inhibitor bafilomycin A1. MHC-I depletion was rescued by bafilomycin A1, but not by lactacystin, indicating that Aβ-mediated MHC-I depletion requires lysosomal activity (Fig. 1I). In the absence of Aβ, lactacystin directly reduced MHC-I levels by inhibiting proteasome activity, generating a peptide important for intracellular MHC-I folding. Because bafilomycin inhibited endocytic lysosomal degradation, the effects of endocytic pathway inhibitors, including the caveolin-dependent endocytosis inhibitor methyl-β-cyclodextrin and the clathrin-dependent endocytosis inhibitor chlorpromazine, were also investigated. Methyl-β-cyclodextrin increased MHC-I levels in the presence or absence of Aβ treatment, whereas chlorpromazine had no effect on MHC-I levels under both conditions (Fig. 1J), suggesting that Aβ enhances MHC-I endocytosis via a caveolin-dependent pathway followed by lysosomal degradation. Together, these observations suggested that the Aβ oligomer has a novel deteriorating function, reducing the stability of MHC-I-β2M complex and stimulating lysosomal degradation of MHC-I.

### Destabilization of MHC-I-β2M complex in AD brains

**Fig. 2:**
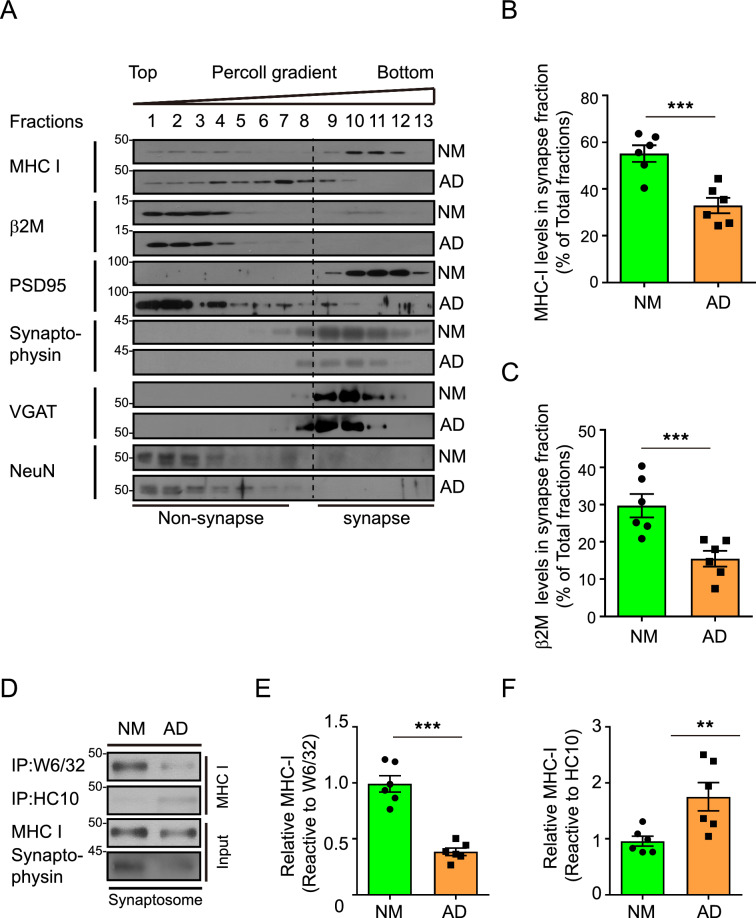
Pathologic changes of neuronal MHC-I–β_2_M complex in the brains of AD patients. **A**–**C** Immunoblot analyses of MHC-I, β_2_M, PSD95, synaptophysin, VGAT, and NeuN in subcellular fractions from aged normal and AD brains. The levels of MHC-I (**B**) and β_2_M (**C**) in synaptosomes were normalized to total protein levels in all fractions (n = 6). **D–F** Immunoprecipitation analyses with specific antibodies of MHC-I-β_2_M complex and β_2_M-free MHC-I in synaptosomes purified from aged normal and AD brains (**C**: n = 6). The levels of the purified β_2_M interacting (**E**) and β_2_M-free (**F**) forms of MHC-I in synaptosomes were normalized to total protein levels. Data are presented as the mean ± standard error of the mean (SEM) (N.S., not significant; *P < 0.05, **P < 0.001 by unpaired two-tailed Student’s t-test)

To investigate whether impaired MHC-I-β2M complex was involved in AD pathology, we investigated synaptic MHC-I levels in AD brains because functional neuronal MHC-I is mainly localized to the synapses [26, 27]. Among the hippocampal fractions in normal brains, almost 60% of total MHC-I was in the synaptic fractions (Fig. 2A, B), a finding confirmed by assessing the expression of several synaptic proteins, including synaptophysin, VGAT, and postsynaptic density (PSD)-95. NeuN, a neuronal nuclear protein, localized to the non-synaptic fractions, also confirming the purity of isolated synaptosomes. MHC-I and β2M levels were reduced 50% in synaptic fractions of AD brains (Fig. 2A–C). The synaptic proteins including synaptophysin and PSD95 were also downregulated in AD synapses (Fig. 2A, D), indicating synaptic degeneration in AD brains [28, 29]. Higher PSD95 localization in non-synaptic fraction may reflect the shift in the distribution of PSD95 from post-synapse to other regions as disease progression [28]. Together, these results indicated that synaptic levels of MHC-I and β2M are reduced in AD brains. Using two kinds of MHC-I conformation-specific antibodies, immunoprecipitation analysis demonstrated that most synaptic MHC-I in synaptosomes of control brains was present in W6/32-sensitive MHC-I-β2M complex form (Fig. 2D, E). In AD synaptosomes, however, most synaptic MHC-I was present as HC-sensitive β2M-free MHC-I heavy chains (Fig. 2D, F).

Since MHC-I-β2M complex formation was assisted by peptide-loading complex in the endoplasmic reticulum (ER) [30], we checked whether the destabilization of MHC-I-β2M complex is due to the impairment of complex formation in the ER. There were no changes in AD brains in the levels of proteins composing the ER folding machinery, including ERp57, protein disulfide isomerase (PDI), tapasin, and transporter associated with antigen processing (TAP) (Additional file 1: Fig. S1A, B). In addition, analysis of isolated MHC-I digested with endoglycosidase-H and peptide-n-glycosidase F showed that glycosylation maturation of MHC-I was not altered in AD brains (Additional file 1: Fig. S1C). These results suggest that intracellular folding and glycosylation maturation of MHC-I–β2M complex were not associated with destabilization of MHC-I-β2M complex in AD brains.

### Identification of NCAM1 interactions with synaptic MHC-I–β2M complexes

**Fig. 3:**
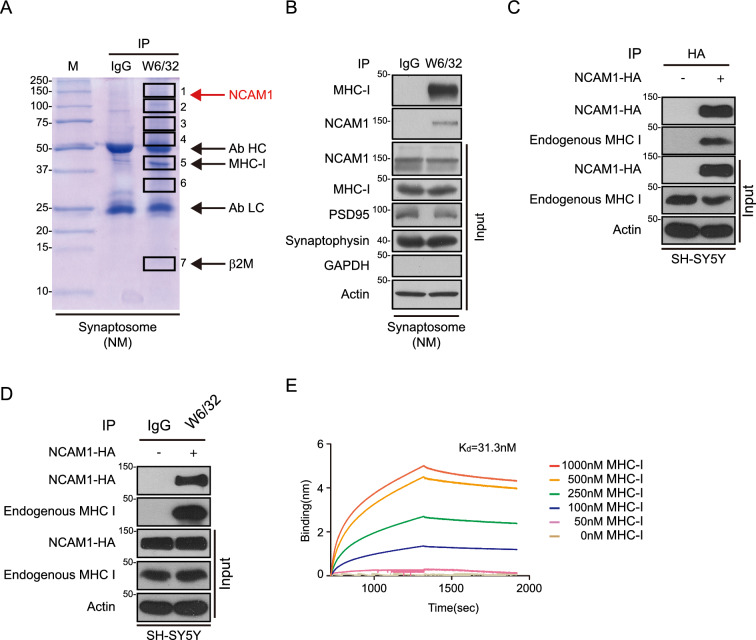
Identification of NCAM1 as a novel neuronal MHC-I interacting protein. **A** Proteins in synaptosomes from normal aged human brain that interacted with MHC-I, as shown by coimmunoprecipitation with W6/32 antibody and staining with Coomassie blue. Gel slices 1–7 were processed by in-gel digestion and LC–MS/MS analysis. **B** Confirmation of the MHC-I–NCAM1 interaction by immunoblotting with antibodies to MHC-I and NCAM1. Synaptosome purity was assessed by immunoblotting with antibodies to PSD95, synaptophysin, and GAPDH. **C**, **D** Coimmunoprecipitation analysis of the interaction between NCAM1 and MHC-I. Lysates of SH-SY5Y cells expressing NCAM1-hemagglutinin (HA) were immunoprecipitated with antibodies to HA (**C**) and W6/32 (**D**), followed by immunoblotting with the anti-HA and anti-MHC-I antibodies (**C**, **D**). The blots are representative of three independent experiments. **E** Bio-Layer Interferometry analysis of the affinity of interaction between MHC-I and NCAM1. NCAM1 protein (100 nM) was immobilized onto a biosensor, which was incubated with recombinant biotinylated MHC-I–β_2_M complex (HLA-A2.1 allele) at concentrations of 0, 50, 100, 250, 500, and 1000 nM. All data are presented as the mean ± SEM (*P < 0.05, **P < 0.001, ***P < 0.005 by unpaired two-tailed Student’s t-test)

Because the structural formation of MHC-I-β2M complex is critical for interaction with its functional counterpart, we hypothesized that a conformational change in MHC-I-β2M complex could alter its interactions with unknown synaptic proteins in AD. To identify proteins that interact with neuronal MHC-I, synaptic proteins binding to MHC-I-β2M complexes were co-purified with W6/32 antibody from the synaptosomes of normal human brain and analyzed with a proteomics approach. In addition to detecting several proteins in synaptosomes previously shown to interact with MHC-I-β2M complex, including GRP78, calnexin, and β-COP [31–33], liquid chromatography-tandem mass spectrometry (LC–MS/MS) identified a 150 kDa protein that interacted with the stable form of MHC-I (Fig. 3A, Additional file 2: Fig. S2A–C, Additional file 3: Table S2). This protein was subsequently identified as NCAM1, a transmembrane glycoprotein that regulates neurite outgrowth, synaptogenesis, and synaptic plasticity [34–37]. This NCAM1-MHC-I interaction was confirmed by immunoblotting of the same synapse fraction with antibody to NCAM1 (Fig. 3B) and by its coimmunoprecipitation from SH-SY5Y cells expressing HA-tagged NCAM1 (Fig. 3C, D). The NCAM1–MHC-I interaction was further confirmed by a bio-layer interferometry assay using recombinant NCAM1 and MHC-I-β2M complex, with binding affinity of K_D_ of 31.3 nM (Fig. 3E). Therefore, NCAM1 was identified as a novel interactor of MHC-I in human brain synaptosome.

### Inhibition of NCAM1 expression and formation of NCAM1–MHC-I complexes in AD

**Fig. 4:**
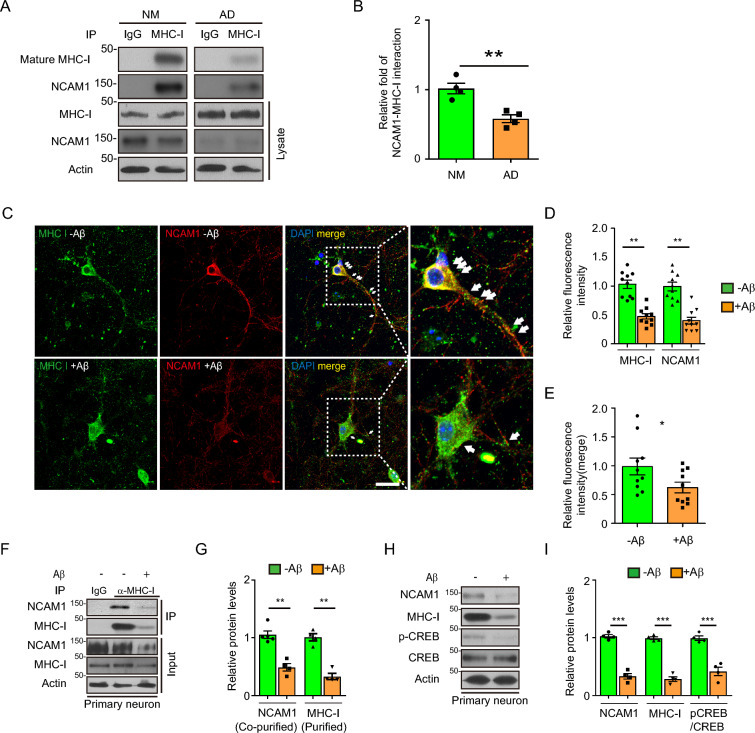
Impaired NCAM1–MHC-I interaction in AD pathology. **A**, **B** Co-immunoprecipitation analysis of interactions between endogenous MHC-I and NCAM1 in the brains of normal subjects (NM) and patients with AD (**A**, **B**; n = 4). The levels of co-immunoprecipitated NCAM1 were normalized to total NCAM1 levels (**B**). The data are presented as the mean ± SEM (**P < 0.001; unpaired two-tailed Student’s t-test). **C**–**E** Co-localization of MHC-I with NCAM1 on primary neurons. Scale bar: 10 µm (**C**). Relative fluorescence intensity of either MHC1 or NCAM1 (**D**) and colocalization of MHC1 and NCAM1 (**F**, **G**). Coimmunoprecipitation analysis of interactions between endogenous MHC-I and NCAM1 in oligomeric Aβ-treated primary neurons (**F**, n = 4). The levels of coimmunoprecipitated NCAM1 and MHC-I were normalized to the levels of total NCAM1 and MHC-I, respectively (**G**). **(H, I)** Oligomeric Aβ-mediated downregulation of NCAM1 signaling detected by CREB phosphorylation (**H**, n = 4). The levels of NCAM1, MHC-I, and phosphorylated CREB were normalized to the levels of total NCAM1, MHC-I, and CREB, respectively (**I**). All data are presented as the mean ± SEM (*P < 0.05, **P < 0.001, ***P < 0.005 by unpaired two-tailed Student’s t-test)

Since NCAM1 is a novel interacting partner of neuronal MHC-I-β2M complex (Fig. 3) and MHC-I-β2M is destabilized by Aβ oligomers and in AD brains (Figs. 1, 2), we speculated that the interaction of NCAM1 with MHC-I-β2M complex is also deregulated. As expected, co-immunoprecipitation assay showed reduction in the amount of NCAM1-MHC-I-β2M complex in AD brains (Fig. 4A, B). In primary neurons, NCAM1 co-localized with MHC-I at the surface of the soma or dendrites, however Aβ oligomer reduced the co-localization intensity between MHC-I and NCAM1 or each protein levels (Fig. 4C–E). Aβ treatment reduced the amount of NCAM1 interacting with MHC-I by about 50% relative to control in primary neurons (Fig. 4F, G). Aβ treatment also reduced CREB phosphorylation, which is part of the final cascade of NCAM1 signaling (Fig. 4H, I), suggesting that the MHC-I–β2M complex is important for interacting with NCAM1 and activating its signaling. Together, these observations suggested that Aβ-mediated destabilization of MHC-I-β2M complex eventually impairs the formation of NCAM1-MHC-I-β2M complex, subsequently dysregulating NCAM1 signaling.

### Stabilization of MHC-I-β2M complex rescued Aβ-mediated MHC-I depletion and dysregulation of NCAM1-MHC-I interactions

**Fig. 5:**
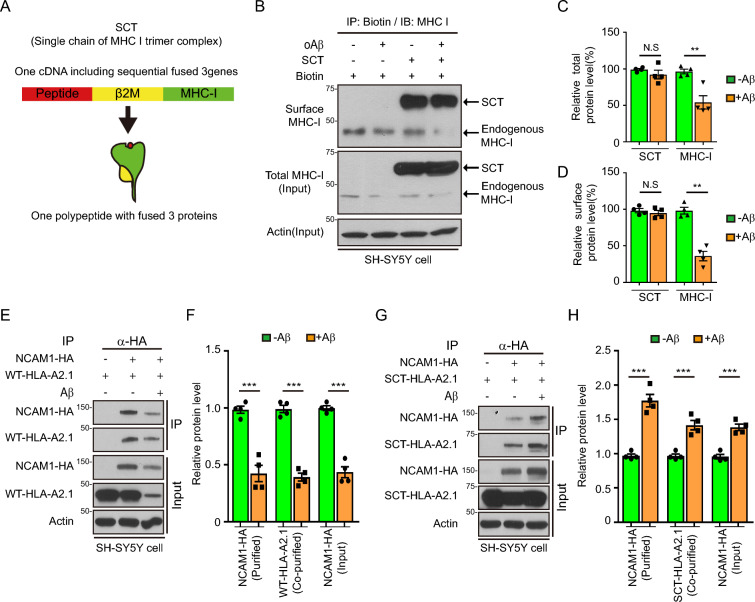
Aβ-resistance of MHC-I increased the interaction of NCAM1–MHC-I. **A** Schematic representation of the structure of the single-chain trimer (SCT). The SCT construct was composed of three genes (MHC-I, β_2_M, and antigen peptide), which resulted in the expression of a single protein composed of these three proteins. **B–D** Immunoblot analyses of endogenous MHC-I and exogenous SCT levels in oAβ-treated SH-SY5Y cells. Biotinylated surface MHC-I and SCT were immunoprecipitated with streptavidin and analyzed by immunoblotting (**B**, n = 4). The levels of total (**C**) and surface (**D**) MHC-I and SCT were normalized to the levels of actin. The data were analyzed by unpaired two-tailed Student’s t-test (**C**, **D**). **E–H** Coimmunoprecipitation analysis of exogenous HLA-A2.1–NCAM1-HA (E, F; n = 4) and SCT–NCAM1-HA (G, H; n = 4) interactions in oAβ-treated SH-SY5Y cells. The levels of coimmunoprecipitated NCAM1-HA (F, H), HLA-A2.1 (**F**), and SCT (**H**) were normalized to actin levels, and the data were analyzed by unpaired two-tailed Student’s t-test (**F**, **H**). The data are presented as the mean ± SEM (N.S., not significant; *P < 0.05, **P < 0.001, ***P < 0.005)

**Fig. 6:**
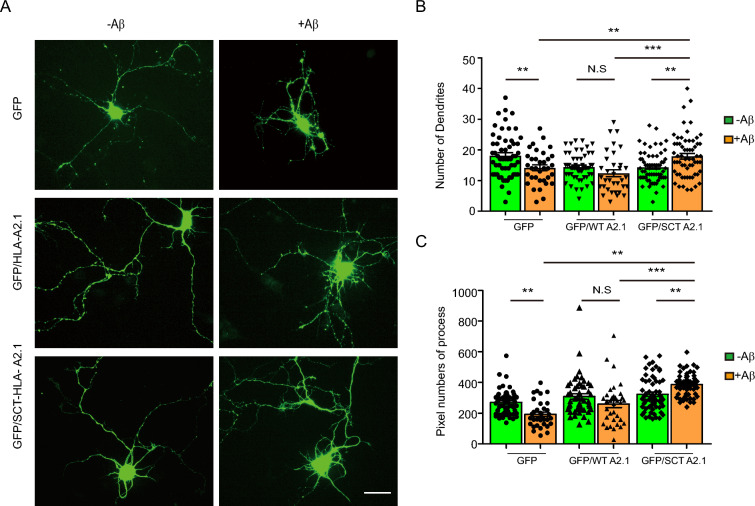
Aβ-resistance of MHC-I inhibited the Aβ-mediated downregulation of NCAM1 signaling. **A**–**C** Representative images of primary neurons expressing green-fluorescent protein (GFP), GFP/HLA-A2.1, and GFP/SCT-HLA-A2.1 in the presence or absence of treatment with oAβ for 8 days in vitro (**A**). Quantification of dendrite numbers (**B**) and the process lengths (**C**) in mock- and Aβ-treated primary neurons expressing GFP, GFP/HLA-A2.1, and GFP/SCT-HLA-A2.1 (n = 3, at least 35 segments per sample; two-way ANOVA with Tukey’s post-hoc comparisons test). Scale bar: 20 µm. The data are presented as the mean ± SEM (N.S., not significant; *P < 0.05, **P < 0.001, ***P < 0.005)

To determine whether inhibition of Aβ-mediated dissociation of MHC-I-β2M complex can rescue downregulated NCAM1 signaling through a recovery of interactions between MHC-I and NCAM1, we utilized a single-chain trimer (SCT) of MHC-I as an Aβ-resistant MHC-I-β2M complex. SCT, a synthetic gene encoding a sequential link of three cDNAs with peptide, β2M, and MHC-I heavy chains (HLA-A2.1 allele) (Fig. 5A) [38], was expressed on the cell surface of SH-SY5Y cells (Fig. 5B). Treatment of SH-SY5Y cells expressing the SCT form of MHC-I-β2M complex with Aβ had no effect on the surface and total levels of expression of SCT. This treatment, however, reduced the total and surface expression of endogenous MHC-I (Fig. 5B–D), demonstrated that the SCT form was resistant to destabilization by Aβ. In addition, Aβ-resistant SCT expression was able to protect MHC-I-NCAM1 interaction from the effects of Aβ treatment. Co-immunoprecipitation assays showed that levels of HLA-A2.1-SCT were resistant to Aβ treatment, resulting in a 1.5-fold increase in complexes of HLA-A2.1-SCT and NCAM1-HA (Fig. 5G, H). However, the HLA-A2.1-WT(wild-type) form was sensitive to Aβ and unable to interact with NCAM1-HA (Fig. 5E, F). Importantly, Aβ-mediated NCAM1 depletion was inhibited by expression of HLA-A2.1-SCT but not by HLA-A2.1-WT (Fig. 5E–H), suggesting that Aβ-mediated NCAM1 depletion depended on the Aβ-resistance of MHC-I–β_2_M complexes. To investigate whether the enhanced SCT-NCAM1 interaction can augment NCAM1 signaling for neurite outgrowth when treated with Aβ, primary neurons co-expressing green-fluorescent protein (GFP) and HLA-A2.1-WT or HLA-A2.1-SCT were treated with vehicle or Aβ (Fig. 6A), and NCAM1 signaling activity was assessed by measuring dendrite number and total length of neuronal processes of GFP-positive neurons after eight divisions. Compared with vehicle treatment, Aβ treatment reduced dendrite numbers and lengths of neuronal processes in GFP expressing neurons (Fig. 6B, C). Notably, HLA-A2.1-SCT expression increased neurite density compared with HLA-A2.1-WT expression and GFP control when treated with Aβ, whereas HLA-A2.1-WT expression did not affect neuronal growth under the same conditions (Fig. 6A–C). These results suggested that the enhanced stability of the MHC-I-β2M complex ameliorated the Aβ-mediated downregulation of NCAM1 signaling in synaptic outgrowth.

## Discussion

The present study revealed that Aβ oligomers destabilizes synaptic MHC-I-β2M complex, impairing the interaction with NCAM-1, a novel MHC-I partner. We here further showed that inhibiting the Aβ-induced destabilization of MHC-I complex by SCT can reverse the neuronal signaling impairment, harnessing a novel therapeutic strategy.

Although studies have been made about the role of neuronal MHC-I in synaptic plasticity [6, 7, 39, 40], little was known about MHC-I function in neurological diseases. Especially, the connection between MHC-I function and its role in AD remained unclear, despite genetic analyses showing an increased frequency of some MHC-I alleles in individuals with AD [10]. We here found that the unstable β2M-free MHC-I heavy chain was increased in AD brains (Fig. 2). The oligomeric Aβ was largely responsible for the destabilization of neuronal MHC-I-β2M complexes (Fig. 1). Aβ treatment caused MHC-I-β2M complex to degenerate into soluble β2M and β2M-free MHC-I heavy chain, leading to rapid internalization of MHC-I heavy chain followed by lysosomal degradation (Fig. 1). This β2M-free MHC-I heavy chain was also found to regulate synapse density negatively in glutamatergic neurons [7], which may be related with synapse loss in AD brains. MHC-I was also recently reported to be involved in the pathogenic mechanisms of the strongest risk factor of sporadic AD, apolipoprotein-E4 [41].

Aβ oligomers induced extracellular release of β2M which was dissociated from surface MHC-I-β2M complex (Fig. 1E, H). Several proteomics analyses have identified β2M as a potential biomarker in the CSF of patients with AD [42, 43]. Interestingly, β2M is a pro-aging factor that impairs cognitive function and neurogenesis, implying relations with AD [44]. β2M could induce cognitive decline via neuroinflammation through toll-like receptor 4 (TLR4) [45]. Hence, the increased release of β2M downstream from Aβ-induced dissociation of MHC-I-β2M complex (Fig. 1) could be related with neuroinflammation and neurodegeneration in AD. How Aβ oligomer dissociates MHC-I-β2M complex is yet uncertain. Aβ oligomer might dissociate MHC-I complex directly or indirectly via unknown mechanisms. Leukocyte immunoglobulin like receptor B2 (LilrB2) is a well-known binding partner with MHC-I [46]. Aβ oligomers bind to LilrB2 [18], which might be a way of dissociating MHC-I-β2M complex indirectly. LilrB2 has a binding preference to β2M-free MHC-I heavy chains rather than the MHC-I-β2M complex in vitro [47]. Additional studies are required to assess the detailed mechanism of MHC-I dissociation and toxic effects of Aβ-mediated extracellular β2M.

Because the function of MHC-I is mainly dependent on the protein with which it interacts, the identification of these proteins interacting with neuronal MHC-I is important for understanding MHC-I function in the CNS [3, 7]. We found here that NCAM1 protein is a novel functional receptor of MHC-I in the CNS (Fig. 3). NCAM1 is expressed in the adult CNS, is highly conserved in vertebrates, and is important for neurite outgrowth and synaptic plasticity in the hippocampus [48, 49]. Aβ decreased the interaction of NCAM1 with MHC-I, and NCAM1 downstream signaling (Fig. 4). Further investigation on the mechanism of NCAM decrease induced by Aβ oligomers is required to elucidate role of MHC-I in NCAM stability. While independent effect of Aβ oligomer on NCAM decrease is also possible, SCT-mediated MHC-I stabilization rescue of decreased NCAM levels and restoring synaptic levels suggest MHC-I-NCAM1 interaction role in protecting NCAM1 decrease by Aβ oligomer (Fig. 5). NCAM, being a neuronal marker, is associated in AD. NCAM-deficient mice display reduced exploratory behavior and spatial learning, similar to the symptoms of AD in humans [50]. NCAM levels are lower in frontal and temporal cortex AD patients than control patients [51] and areare increased in serum of AD patients [52]. Aβ mediates the shedding of NCAM2, another neural cell adhesion molecule, leading to loss of glutamatergic synapses [53]. These results uggest that the overall stability of MHC-I is critical for its interaction with NCAM1 and potential role of MHC-I-NCAM1 interaction on pathogenesis of AD.

Various forms and alleles of MHC-I may regulate various synaptic functions via interactions with yet undetermined MHC-I receptors that can distinguish among MHC-I structures, suggesting the difference in regional or individual susceptibility of neurodegeneration in AD. The mechanisms regulating the transition of MHC-I structure and the identification of unidentified MHC-I interaction partners should be investigated further to understand the detailed functions of neuronal MHC-I.

## Conclusion

In summary, we report a novel pathological mechanism of AD, the Aβ-mediated destabilization of the MHC-I-β2M complex and subsequent disruption of NCAM1 signaling. Furthermore, the SCT-mediated restoration of the MHC-I–β2M complex may be a promising therapeutic strategy for restoring synaptic function in AD patients.

### Supplementary Information

**Additional file 1: Figure S1. **Analysis of expression of the peptide-loading complex components and glycosylation maturation of MHC-I in AD. **A**, **B** Immunoblot analyses of the components of the peptide-loading complex (ERp57, PDI, tapasin, and TAP) in aged normal and AD brains (**A**; n = 3 each). **B** The levels of ERp57, PDI, tapasin, and TAP were normalized to those of actin. **C** Intracellular MHC-I was immunoprecipitated from aged normal and AD brains, and digested with endoglycosidase-H (Endo-H) or peptide-n-glycosidase F (PNGase F). Glycosylated and deglycosylated forms of MHC-I were detected by immunoblotting. The data are presented as the mean ± SEM (N.S, not significant; unpaired two-tailed Student’s t-test).

**Additional file 2: Figure S2.** Identification of NCAM1 as an MHC-I interacting protein. **A** Summary of proteins identified as interacting with synaptic MHC-I–β_2_M complex. Proteins were aligned by id frequency. Previously identified MHC-I interacting proteins were based on the BioGRID database and labeled in red. **B** NCAM1 identified by liquid chromatography-tandem mass spectrometry (LC-MS/MS) analysis with 28 exclusive unique peptides and 38 exclusive unique spectra from 77 total spectra with 35% sequence coverage. The yellow-highlighted peptides are those detected by LC-MS/MS analysis. The green-highlighted “M” indicates an oxidated form of methionine. **C** The LC-MS/MS spectrum of “GLGEISAASEFK”, a distinctive and representative peptide of NCAM1. The Y and B ions are displayed as blue and red peaks, respectively, and the green peaks indicate the immonium ions and internal cleaved ions.

**Additional file 3: Table S1. **Characteristics of the subjects assessed in this study. **Table S2. **Summary of top interactome of synaptic MHC-I–β_2_M complex.

## Data Availability

All data generated or analysed during this study are included in this published article.

## Abbreviations

MHC-1: Major histocompatibility complex I
AD: Alzheimer’s disease
Aβ: β-Amyloid
β2M: β2-Microglobulin
NCAM1: Neural cell adhesion molecule 1
CNS: Central nervous system
AMPA: α-Amino-3-hydroxy-5-methyl-4-isoxazolepropionic acid
NMDA: n-methyl-d-aspartate
FACS: Fluorescence-activated cell sorting
LC: Lactacystin
BFA1: Bafilomycin
MβCD: Methyl-β-cyclodextrin
CPZ: Chlorpromazine
ER: Endoplasmic reticulum
SCT: Single-chain trimer
LilrB2: Leukocyte immunoglobulin like receptor B2
