# Supplementary figures and images for: Neuronal MHC-I complex is destabilized by amyloid-β and its implications in Alzheimer’s disease
Source: Cell Biosci. 2023 Sep 29;13:181. doi: 10.1186/s13578-023-01132-1 (PMC10540404; doi:10.1186/s13578-023-01132-1)

A

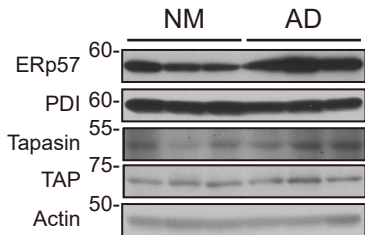

B

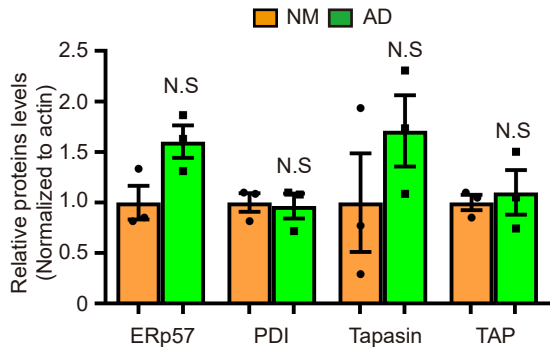

C

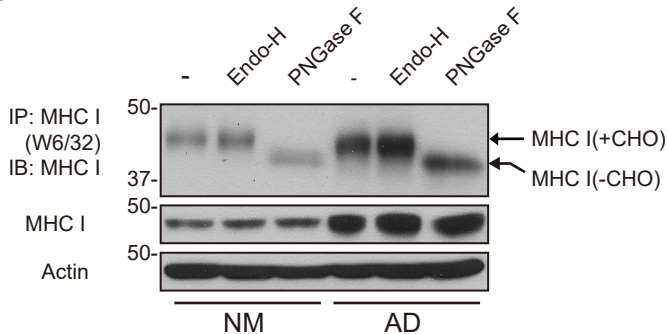

Supplement: Supplementary file 1 — Additional file 1: Figure S1. Analysis of expression of the peptide-loading complex components and glycosylation maturation of MHC-I in AD. A, B Immunoblot analyses of the components of the peptide-loading complex (ERp57, PDI, tapasin, and TAP) in aged normal and AD brains (A; n = 3 each). B The levels of ERp57, PDI, tapasin, and TAP were normalized to those of actin. C Intracellular MHC-I was immunoprecipitated from aged normal and AD brains, and digested with endoglycosidase-H (Endo-H) or peptide-n-glycosidase F (PNGase F). Glycosylated and deglycosylated forms of MHC-I were detected by immunoblotting. The data are presented as the mean ± SEM (N.S, not significant; unpaired two-tailed Student’s t-test). [file 13578_2023_1132_MOESM1_ESM.pdf]
