# Supplementary material for: Neuronal MHC-I complex is destabilized by amyloid-β and its implications in Alzheimer’s disease
Source: Cell Biosci. 2023 Sep 29;13:181. doi: 10.1186/s13578-023-01132-1 (PMC10540404; doi:10.1186/s13578-023-01132-1)

A

Id frequency	Protein
10	NCAM1
8	ATP1A3
6	SLC1A3, SHROOM3
5	DCD, NFASC, PLP1
4	MAP1B, SPTAN1
3	HSP90AA1, SPTBN1, ATP2B1, HBB, HSPA2
2	HSP90B1, DPYSL2, SLC1A2, GPD2, CFL1, LGALS3BP, TG, CLTC, FLG2, CNTN1, LYZ, SV2A, STX1B, CDC5L, CKB
1	HIST1H3A, EIF5AL1, RPS10, RPL31, KSR1 , RFX2, NME7, GOLGB1, COPB1, H2AFJ, DNPH1, RPL23, DSP, JUP, HRNR, DSG1, ATP1A1, NRCAM, CAND1, ARG1, DSC1, L1CAM, ICAM5, TXN, ANXA2, ATP1A2, GAPDH, UBA52, KIF5B, AC1, CSTA, ATOH1, GAS2L2, XP32, VCP, HSPA8, NDUFS1, CADM1, IGSF8, SLC3A2, HSPA1B, ANK2, CANX, HSPA9, XRCC6, HSPA5, MAG, HSPA6, HSP90AB1, TRIM2, PADI2, CD44, XPNPEP1, AP2B1, SLC4A4, SV2B, PRKCSH, C4B, SYN1, IGHG3, KATNAL2, MBP, PGM2L1, ACTB, ATP6V0A1, HSPD1, SYT1, PPP2R1A, CXorf40A, ENO2, LDHB, STX1A, IGHV3OR16-9, MAPRE3, ATP5C1, YWHAE, SH3GL2, DECR1, MDH2, CKM, PPP3CA, NAPA, GPM6A, IGKC, KIF20B, PPIA, CYB5R3, HSPH1, CLU, TOLLIP, SLC25A6, NDRG2, IGHG1, RPS9, ALAD, SYP, GRP78, CLTA, IGHV6-1, PRRT2, EIF5A, PCDH11X, HLA-A, HLA-B, B2M

B

```
MLQTKDLIWT LFFLGTAVSL QVDIVPSQGE ISVGESKFFL CQVAGDAKDK
DISWFSPNGE KLTPNQQRIS VVWNDDSSST LTIYNANIDD AGIYKCVVTG
EDGSESEATV NVKIFQKLMF KNAPTPQEFR EGEDAVIVCD VVSSLPPTII
WKHKGRDVIL KKDVRFIVLS NNYLQIRGIK KTDEGTYRCE GRILARGEIN
FKDIQVIVNV PPTIQARQNI VNATANLGQS VTLVCDAEGF PEPTMSWTKD
GEQIEQEEDD EKYIFSDDSS QLTIKKVDKN DEAEYICIAE NKAGEQDATI
HLKVFAKPKI TYVENQTAME LEEQVTLTCE ASGDPIPSIT WRTSTRNISS
EEKASWTRPE KQETLDGHMV VRSHARVSSL TLKSIQYTDA GEYICTASNT
IGQDSQSMYL EVQYAPKLQG PVAVYTWEGN QVNITCEVFA YPSATISWFR
DGQLLPSSNY SNIKIYNTPS ASYLEVTPDS ENDFGNYNCT AVNRIGQESL
EFILVQADTP SSPSIDQVEP YSSTAQVQFD EPEATGGVPI LKYKAEWRAV
GEEVWHSKWY DAKEASMEGI VTIVGLKPET TYAVRLAALN GKGLGEISAA
SEFKTQPVQG EPSAPKLEGQ MGEDGNSIKV NLIKQDDGGS PIRHYLVRYR
ALSSEWKPEI RLPSGSDHVM LKSLDWNAEY EVYVVAENQQ GKSKAAHFVF
RTSAQPTAIP ANGSPTSGLS TGAIVGILIV IFVLLLVVVD ITCYFLNKCG
LFMCIAVNLC GKAGPGAKGK DMEEGKAAFS KDESKEPIVE VRTEEERTPN
```

C

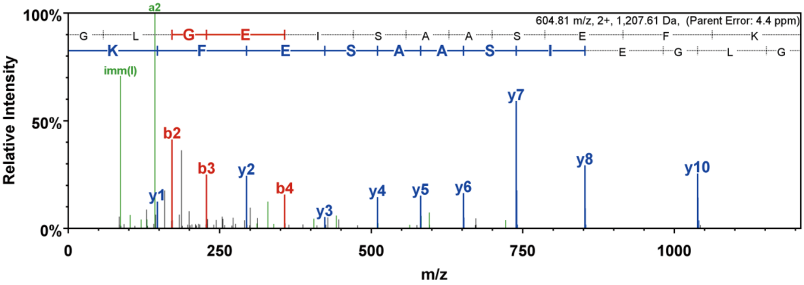

Supplement: Supplementary file 2 — Additional file 2: Figure S2. Identification of NCAM1 as an MHC-I interacting protein. A Summary of proteins identified as interacting with synaptic MHC-I–β2M complex. Proteins were aligned by id frequency. Previously identified MHC-I interacting proteins were based on the BioGRID database and labeled in red. B NCAM1 identified by liquid chromatography-tandem mass spectrometry (LC-MS/MS) analysis with 28 exclusive unique peptides and 38 exclusive unique spectra from 77 total spectra with 35% sequence coverage. The yellow-highlighted peptides are those detected by LC-MS/MS analysis. The green-highlighted “M” indicates an oxidated form of methionine. C The LC-MS/MS spectrum of “GLGEISAASEFK”, a distinctive and representative peptide of NCAM1. The Y and B ions are displayed as blue and red peaks, respectively, and the green peaks indicate the immonium ions and internal cleaved ions. [file 13578_2023_1132_MOESM2_ESM.pdf]
