# Supplementary material for: Neuronal MHC-I complex is destabilized by amyloid-β and its implications in Alzheimer’s disease
Source: Cell Biosci. 2023 Sep 29;13:181. doi: 10.1186/s13578-023-01132-1 (PMC10540404; doi:10.1186/s13578-023-01132-1)
Supplement: Supplementary file 3 — Additional file 3: Table S1. Characteristics of the subjects assessed in this study. Table S2. Summary of top interactome of synaptic MHC-I–β2M complex. [file 13578_2023_1132_MOESM3_ESM.docx]

**Supplementary Information**

**Supplementary Table 1. Characteristics of the subjects assessed in this study**

| **Diagnosis** | **Sex** | **Age, yr** | **Braak** | **Amyloid** | **PMD** |
| --- | --- | --- | --- | --- | --- |
| Alzheimer's disease | M | 85 | 5 | C | 07:10 |
| Alzheimer's disease | M | 65 | 6 | C | 08:50 |
| Alzheimer's disease | M | 65 | 5 | C | 05:50 |
| Alzheimer's disease | M | 65 | 5 | C | 07:20 |
| Alzheimer's disease | M | 87 | 5 | C | 06:10 |
| Alzheimer's disease | M | 67 | 5 | C | 04:10 |
| Alzheimer's disease | M | 70 | 6 | C | 04:50 |
| Alzheimer's disease | M | 82 | 5 | C | 05:15 |
| Nondemented control | M | 73 | 0 | O | 24:45 |
| Nondemented control | M | 71 | 1 | O | 07:40 |
| Nondemented control | M | 87 | 1 | A | 10:20 |
| Nondemented control | M | 80 | 0 | O | 07:15 |
| Nondemented control | M | 84 | 1 | A | 05:35 |
| Nondemented control | M | 82 | 1 | O | 05:10 |
| Nondemented control | M | 78 | 1 | O | <17:40 |

**Supplementary Table 2. Summary of top interactome of synaptic MHC-I–β_2_M complex**

| **Summary of Top 10 interactome of synaptic MHC-I–β_2_M complex (Gel slice 1)** | | | | | | | | | | | | | |
| --- | --- | --- | --- | --- | --- | --- | --- | --- | --- | --- | --- | --- | --- |
| **Symbol**  **(accession no.)** | **Gene name** | | **No. of proteins** | | **Unique peptides** | | **Unique sequence coverage(%)** | | **Score** | | **Location** | | **Function** |
| CLTC  (Q00610) | Clathrin heavy chain 1 | | 7 | | 8 | | 6.8 | | 73.909 | | Cytoplasm | | Endocytosis |
| NFASC  (H7BY57) | Neurofascin | | 4 | | 5 | | 5.7 | | 40.802 | | Plasma membrane | | Cell adhesion |
| ATP1A3  (M0R116) | Sodium/potassium-transporting ATPase subunit alpha | | 11 | | 4 | | 5.4 | | 27.088 | | Plasma membrane | | [Transport](https://www.uniprot.org/keywords/KW-0813) |
| NCAM1  (A0A087WTF6) | Neural cell adhesion molecule 1 | | 12 | | 2 | | 3.2 | | 26.763 | | Plasma membrane | | Cell adhesion |
| ATP2B1  (E7ERY9) | Calcium-transporting ATPase | | 7 | | 2 | | 3.8 | | 15.128 | | Plasma membrane | | [Transport](https://www.uniprot.org/keywords/KW-0813) |
| MAP1B  (P46821) | Microtubule-associated protein 1B  (fragment) | | 4 | | 2 | | 1 | | 12.462 | | Cytoplasm | | Autophagy |
| FLG2  (Q5D862) | Filaggrin-2 (fragment) | | 3 | | 2 | | 11 | | 10.886 | | Cytoplasm | | Calcium binding |
| TG  (P01266) | Thyroglobulin (fragment) | | 4 | | 1 | | 13.4 | | 8.1104 | | Extracellular | | Hormone |
| GOLGB1  (H0Y867) | Golgin subfamily B member 1 (fragment) | | 4 | | 2 | | 1 | | 7.9462 | | Golgi | | [Transport](https://www.uniprot.org/keywords/KW-0813) |
| COPB1  (E9PP73) | Coatomer subunit beta | | 2 | | 1 | | 8.5 | | 5.9606 | | Cytoplasm | | [Transport](https://www.uniprot.org/keywords/KW-0813) |
| **Summary of Top 10 interactome of synaptic MHC-I–β_2_M complex (Gel slice 2)** | | | | | | | | | | | | | |
| **Symbol**  **(accession no.)** | | **Gene name** | | **No. of proteins** | | **Unique peptides** | | **Unique sequence coverage(%)** | | **Score** | | **Location** | **Function** |
| SPTBN1  (Q01082) | | Spectrin beta chain, non-erythrocytic 1  (fragment) | | 6 | | 8 | | 4.2 | | 56.482 | | Cytoplasm | [Actin-binding](https://www.uniprot.org/keywords/KW-0009) |
| ATP1A3  (M0R116) | | Sodium/potassium-transporting ATPase subunit alpha | | 7 | | 3 | | 4.3 | | 24.738 | | Plasma membrane | [Transport](https://www.uniprot.org/keywords/KW-0813) |
| NFASC  (H7BY57) | | Neurofascin | | 3 | | 2 | | 2.7 | | 18.825 | | Plasma membrane | Cell adhesion |
| SHROOM3  (Q8TF72) | | Protein Shroom3 | | 1 | | 1 | | 0.5 | | 12.89 | | Cytoplasm | [Actin-binding](https://www.uniprot.org/keywords/KW-0009) |
| NCAM1  (H7BYX6) | | Neural cell adhesion molecule 1 | | 12 | | 2 | | 2.9 | | 12.176 | | Plasma membrane | Cell adhesion |
| SLC1A2  (C9J9N5) | | Amino acid transporter | | 1 | | 2 | | 26.5 | | 11.659 | | Nucleus | [Transport](https://www.uniprot.org/keywords/KW-0813) |
| CNTN1  (Q12860) | | Contactin-1 | | 4 | | 1 | | 13.4 | | 10.549 | | Plasma membrane | Cell adhesion |
| SLC1A2  (C9J9N5) | | Excitatory amino acid transporter 2 | | 2 | | 1 | | 7.5 | | 10.013 | | Plasma membrane | [Transport](https://www.uniprot.org/keywords/KW-0813) |
| AP2B1 (A0A087WYD1) | | AP-2 complex subunit beta | | 2 | | 1 | | 15.6 | | 9.3179 | | Cytoplasm | [Endocytosis](https://www.uniprot.org/keywords/KW-0648) |
| TG  (P01266) | | Thyroglobulin (fragment) | | 11 | | 1 | | 31.5 | | 8.3048 | | Extracellular | Hormone |

| **Summary of Top 10 interactome of synaptic MHC-I–β_2_M complex (Gel slice 3)** | | | | | | | |
| --- | --- | --- | --- | --- | --- | --- | --- |
| **Symbol**  **(accession no.)** | **Gene name** | **No. of proteins** | **Unique peptides** | **Unique sequence coverage(%)** | **Score** | **Location** | **Function** |
| SPTAN1  (Q13813) | Spectrin alpha chain (fragment) | 3 | 3 | 1.3 | 323.31 | Cytoplasm | [Actin-binding](https://www.uniprot.org/keywords/KW-0009) |
| NCAM1  (A0A087WX77) | Neural cell adhesion molecule 1  (fragment) | 4 | 2 | 2.2 | 262.96 | Plasma membrane | Cell adhesion |
| ATP1A3  (M0R116) | Sodium/potassium-transporting ATPase subunit alpha (fragment) | 4 | 6 | 8.3 | 205.06 | Plasma membrane | [Transport](https://www.uniprot.org/keywords/KW-0813) |
| DSP  (P15924) | Desmoplakin (fragment) | 1 | 22 | 9.4 | 142.19 | Plasma membrane | Cell adhesion |
| SPTBN1  (Q01082) | Spectrin beta chain (fragment) | 3 | 15 | 8 | 100.18 | Plasma membrane | [Actin-binding](https://www.uniprot.org/keywords/KW-0009) |
| JUP  (P14923) | Junction plakoglobin | 7 | 11 | 19.3 | 76.222 | Plasma membrane | Cell adhesion |
| HRNR  (Q86YZ3) | Hornerin (fragment) | 2 | 11 | 16 | 71.418 | Plasma membrane | [Calcium binding](https://www.ebi.ac.uk/QuickGO/term/GO:0005509) |
| DSG1  (Q02413) | Desmoglein-1 (fragment) | 1 | 9 | 13.7 | 61.798 | Plasma membrane | Cell adhesion |
| CNTN1  (Q12860) | Contactin-1 (fragment) | 2 | 9 | 10.9 | 55.565 | Plasma membrane | Cell adhesion |
| SV2A  (Q7L0J3) | Synaptic vesicle glycoprotein 2A | 4 | 7 | 8.8 | 55.203 | Plasma membrane | [Synaptic](https://www.uniprot.org/keywords/KW-0813) transmission |

| **Summary of Top 10 interactome of synaptic MHC-I–β_2_M complex (Gel slice 4)** | | | | | | | |
| --- | --- | --- | --- | --- | --- | --- | --- |
| **Symbol**  **(accession no.)** | **Gene name** | **No. of proteins** | **Unique peptides** | **Unique sequence coverage(%)** | **Score** | **Location** | **Function** |
| HSP90B1 (P14625) | Endoplasmin | 5 | 5 | 7.1 | 33.764 | Endoplasmic reticulum | [Chaperone](https://www.uniprot.org/keywords/KW-0143) |
| CANX  (P27824) | Calnexin | 12 | 4 | 8.9 | 29.857 | Endoplasmic reticulum | Chaperone |
| VCP  (P55072) | Transitional endoplasmic reticulum ATPase | 1 | 3 | 4.2 | 25.139 | Cytoplasm | [Transport](https://www.uniprot.org/keywords/KW-0813) |
| ANK2  (Q01484) | Ankyrin-2 | 14 | 4 | 7.9 | 21.854 | Plasma membrane | [Transport](https://www.uniprot.org/keywords/KW-0813) |
| GRP78  (P11021) | 78 kDa glucose-regulated protein | 1 | 3 | 4.2 | 20.139 | Endoplasmic reticulum | Chaperone |
| TRIM2  (Q9C040 | Tripartite motif-containing protein 2 | 18 | 4 | 5.9 | 19.834 | Cytoplasm | Ubiquitination |
| HSPA8  (P11142) | Heat shock cognate 71 kDa protein | 13 | 10 | 20.7 | 14.91 | Cytoplasm | Chaperone |
| LGALS3BP  (Q08380) | Galectin-3-binding protein | 10 | 10 | 20.7 | 12.17 | Extracellular | Cell adhesion |
| HSP90AA1 (P07900) | Heat shock protein HSP 90-alpha (fragment) | 6 | 8 | 10.9 | 11.82 | Cytoplasm | Chaperone |
| PADI2  (Q9Y2J8) | Protein-arginine deiminase type-2 | 16 | 5 | 12.4 | 8.52 | Cytoplasm | [Hydrolase](https://www.uniprot.org/keywords/KW-0378) |

| **Summary of Top 10 interactome of synaptic MHC-I–β_2_M complex (Gel slice 5)** | | | | | | | |
| --- | --- | --- | --- | --- | --- | --- | --- |
| **Symbol**  **(accession no.)** | **Gene name** | **No. of proteins** | **Unique peptides** | **Unique sequence coverage(%)** | **Score** | **Location** | **Function** |
| GPD2  (P43304) | Glycerol-3-phosphate dehydrogenase (fragment) | 2 | 29 | 50.9 | 274.23 | Mitochondria | [Oxidoreductase](https://www.uniprot.org/keywords/KW-0560) |
| HLA-B  (P01889) | HLA class I histocompatibility antigen, B-7 alpha chain | 3 | 10 | 20 | 200.47 | Plasma membrane | Antigen presentation |
| HLA-A  (P01892) | HLA class I histocompatibility antigen, A-2 alpha chain | 3 | 16 | 30 | 169.79 | Plasma membrane | Antigen presentation |
| CD44  (H0YD13) | CD44 antigen | 10 | 14 | 14.1 | 140.22 | Plasma membrane | Cell adhesion |
| SPTAN1 (A0A0D9SF54) | Spectrin alpha chain (fragment) | 4 | 18 | 9 | 120.7 | Cytoplasm | [Actin-binding](https://www.uniprot.org/keywords/KW-0009) |
| CADM1 (A0A087X0T8) | Cell adhesion molecule 1 (fragment) | 11 | 4 | 16.2 | 93.21 | Plasma membrane | Cell adhesion |
| IGHG3  (A0A075B6N8) | Ig gamma-3 chain C region (fragment) | 15 | 12 | 16.2 | 92.933 | Extracellular | Antibody |
| PGM2L1  (Q6PCE3) | Glucose 1,6-bisphosphate synthase  (fragment) | 2 | 12 | 6.6 | 83.788 | Cytoplasm | Transferase |
| MAP1B  (P46821) | Microtubule-associated protein 1B (fragment) | 5 | 8 | 4.3 | 56.739 | Cytoplasm | Autophagy |
| IGSF8  (Q969P0) | Immunoglobulin superfamily member 8 (fragment) | 2 | 8 | 16.2 | 53.59 | Plasma membrane | [Cell proliferation](https://www.ebi.ac.uk/QuickGO/term/GO:0008283) |

| **Summary of Top 10 interactome of synaptic MHC-I–β_2_M complex (Gel slice 6)** | | | | | | | |
| --- | --- | --- | --- | --- | --- | --- | --- |
| **Symbol**  **(accession no.)** | **Gene name** | **# of proteins** | **Unique peptides** | **Unique sequence coverage(%)** | **Score** | **Location** | **Function** |
| DPYSL2  (Q16555) | Dihydropyrimidinase-related protein 2 (fragment) | 4 | 11 | 26.9 | 106.33 | Cytoplasm | [Neurogenesis](https://www.uniprot.org/keywords/KW-0524) |
| HSPD1  (P10809) | 60 kDa heat shock protein, mitochondrial (fragment) | 6 | 5 | 8.9 | 100.5 | Mitochondria | Chaperone |
| SYT1  (J3KQA0) | Synaptotagmin I, isoform CRA_b (fragment) | 3 | 5 | 11.9 | 50.824 | Plasma membrane | [synaptic transmission](https://www.ebi.ac.uk/QuickGO/term/GO:0007268) |
| MAP1B (D6RA32) | Microtubule-associated protein 1B (fragment) | 4 | 5 | 10.7 | 36.611 | Cytoplasm | Autophagy |
| DCD  (P81605) | Dermcidin (fragment) | 1 | 2 | 20 | 17.631 | Extracellular | [Protease](https://www.uniprot.org/keywords/KW-0645) |
| SLC1A2 (C9J9N5) | **Excitatory amino acid transporter 2**  (fragment) | 2 | 1 | 7.5 | 13.744 | Plasma membrane | [Transport](https://www.uniprot.org/keywords/KW-0813) |
| HSP90AA1 (P07900) | Heat shock protein HSP 90-alpha (fragment) | 5 | 2 | 3.7 | 13.376 | Cytoplasm | Chaperone |
| GPD2  (P43304) | Glycerol-3-phosphate dehydrogenase, mitochondrial (fragment) | 1 | 1 | 2.5 | 13.01 | Mitochondria | [Oxidoreductase](https://www.uniprot.org/keywords/KW-0560) |
| PPP2R1A (C9J9C1) | Serine/threonine-protein phosphatase 2A 65 kDa regulatory subunit A alpha isoform (fragment) | 2 | 1 | 2.5 | 8.8735 | Plasma membrane | [Chromosome partition](https://www.uniprot.org/keywords/KW-0159) |

| **Summary of Top 10 interactome of synaptic MHC-I–β2M complex (Gel slice 7)** | | | | | | | |
| --- | --- | --- | --- | --- | --- | --- | --- |
| **Symbol**  **(accession no.)** | **Gene name** | **No. of proteins** | **Unique peptides** | **Unique sequence coverage(%)** | **Score** | **Location** | **Function** |
| CXorf40A (Q8TE69) | Protein CXorf40A | 8 | 11 | 38 | 178.5 | Unknown | Unknown |
| CKB  (P12277) | Creatine kinase B-type (fragment) | 4 | 8 | 25.2 | 89.731 | Cytoplasm | [Kinase](https://www.uniprot.org/keywords/KW-0418) |
| ENO2  (P09104) | Gamma-enolase (fragment) | 12 | 8 | 32 | 73.934 | Plasma membrane | [Glycolysis](https://www.uniprot.org/keywords/KW-0324) |
| LDHB  (P07195) | L-lactate dehydrogenase B chain (fragment) | 11 | 7 | 23.4 | 56.437 | Cytoplasm | [Oxidoreductase](https://www.uniprot.org/keywords/KW-0560) |
| HBB  (P68871) | Hemoglobin subunit beta | 10 | 3 | 27.9 | 51.86 | Extracellular | [Transport](https://www.uniprot.org/keywords/KW-0813) |
| EIF5AL1  (Q6IS14) | Eukaryotic translation initiation factor 5A-1-like | 2 | 1 | 15.6 | 48.15 | Nucleus | [Protein biosynthesis](https://www.uniprot.org/keywords/KW-0648) |
| STX1A  (Q16623) | Syntaxin-1A (fragment) | 4 | 4 | 18.8 | 42.421 | Cytoplasm | [synaptic transmission](https://www.ebi.ac.uk/QuickGO/term/GO:0007268) |
| B2M  (P61769) | Beta-2-microglobulin | 5 | 1 | 19.8 | 42.07 | Extracellular | Antigen presentation |
| CFL1  (G3V1A4) | Cofilin 1 | 4 | 1 | 13.4 | 40.11 | Cytoplasm | [Actin-binding](https://www.uniprot.org/keywords/KW-0009) |
| ATP5C1  (P36542) | ATP synthase subunit gamma, mitochondrial (fragment) | 1 | 6 | 21.1 | 39.218 | Mitochondria | [Transport](https://www.uniprot.org/keywords/KW-0813) |
